# Supplementary material for: Enhanced-Precision Measurement of Glutathionyl Hemoglobin by MALDI-ToF MS
Source: Molecules. 2023 Jan 4;28(2):497. doi: 10.3390/molecules28020497 (PMC9863390; doi:10.3390/molecules28020497)
Supplement: Supplementary file 1 [file molecules-28-00497-s001.zip › Supplementary Material File S1.pdf]

Enhanced-precision measurement of glutathionyl hemoglobin by MALDI-ToF MS

Federico Maria Rubino, .

molecules-xxxxx (2022)

Supplementary Material File S1. Operative checklist for the measurement of glutathionyl hemoglobin in human red blood cells.

General information.

1 Safety notes.

Equipment. All equipment that involves occupational hazard should be in proper operating conditions. Electrical appliances should conform to local regulations and be properly plugged in the sockets to avoid electrical shock and mechanical hurt. Pipetting and other liquid transfer should be performed with properly liquid-tight hardware and disposable plasticware to avoid unnecessary dispersion of reagents and biological samples.

Reagents. All reagents used in this method have been selected for minimal toxicity and absence of carcinogenic activity, as far as current toxicological knowledge allows. Organic solvent and their mixtures with water and with samples are not flammable. Acidic and caustic reagents should be prepared in the minimum amount and handled with proper precautions.

Samples. Trained medical personnel only, wearing appropriate protections, should perform blood sample withdrawal from subjects, under appropriate safety conditions. Blood samples should be handled in the laboratory with due precaution as referring to their origin and possible hazards. After protein precipitation, biological risk is mitigated.

Personal protective equipment. Perform blood fractionation and analysis when wearing the necessary laboratory attire and personal protective equipment. Latex or nitrile disposable gloves, face mask and shield should be worn during the entire procedure. Caution should be exercised to avoid contact of gloved hands with unprotected computer keyboards.

Waste management. The method has been developed to minimize the use of disposable plasticware by planning the number of samples for each lot of analyses. Reagents should be prepared in the appropriate minimum quantities to avoid waste. Spent sample plates should be discarded according to local regulations.

2 General equipment.

a) Cold storage and transport facilities (+4°C; -20°C).
b) Clinical hemocromocytometer to measure hematological parameters in fresh blood samples.
c) Clinical centrifuge to separate plasma from red blood cells.
d) Vortex-type tube shaker for sample-matrix mixing in Eppendorf plastic mini-tubes
e) Mini-centrifuge for Eppendorf plastic mini-tubes for final MALDI sample preparation.
f) Single-channel pipettors with disposable tips: 1,000, 200, 20 and 2.0 μL
g) Plate reader for spectrophotometric measurements at 450 and 540nm. The employed model uses optical filters at 450 nm (filter bandpass 8.5-16 nm) for hemoglobin measurement.
h) Computer and data-transfer devices for data storage and elaboration with spreadsheet.

3 General glass and plasticware.

Glassware

Dark glass 50- and 100-mL stoppered bottles for stock solution preparations.

Disposable glassware

Vacutainer-type tubes for blood sampling (anticoagulant choice not critical)

Disposable plasticware

Eppendorf-type tubes for in-field blood fractionation with snap-cap or screw-cap (2-mL, 0.5 mL)

Polypropylene tubes with stopcocks (10-mL)
Pipettor disposable tips: 1,000, 200, 20 and 2 µL
Polystyrene reading plates, 96 wells, 250 µL well volume, with lid
Tight-close plastic sample bags
Sanitary box for biological waste disposal
Sanitary box for sharps disposal

4 Reagents.

Deionized water from MilliQ system
Acetonitrile, chromatography grade (any of several brands)
Trifluoroacetic Acid, (Sigma-Aldrich, 80457, eluent additive for LC-MS, LiChropur)
Sinapinic Acid, special MALDI grade (Sigma-Aldrich 85429-1G)
Hemoglobin, human (Sigma-Aldrich, H7379, lyophilized powder)

Procedure for the fractionation of whole blood samples.

a) Blood samples (approx. 3 mL) are obtained by venipuncture in 5-mL vacutainer-type tubes.
b) Rapid cooling to sub-ambient temperature can be achieved in a non-freezing ice-water mixture if samples stand for more than one hour before fractionation. Complete blood fractionation should be performed the soonest possible.
c) From the cooled whole blood sample withdraw a 200- µL whole blood sample in a 0.5 mL Eppendorf cone for hematocrit measurement. Store at +4°C and perform measurement within 6-12 h.
d) Centrifuge the remaining whole blood sample (12,000 rpm, 10 min, +4°C) to separate plasma and red blood cells.
e) Separate plasma in appropriate volume aliquots as follows, without disturbing the settled red blood cells.
   a. 2 x 0.5 mL in 1.5-mL Eppendorf tubes
   b. 1 x remaining plasma volume in one 2.0-mL Eppendorf tube
f) Separate the red blood cells as following.
   a. 4 x 50 µL aliquots of red blood cells in 0.5-mL Eppendorf tubes
   b. 1 x remaining red blood cells in one 2.0-mL Eppendorf tube
g) Place sampled aliquots and reference leaflet in plastic bag for fast cooling at -20°C.

Discard waste in the appropriate containers.

Hemocytometric characterization of blood samples

Total hemoglobin concentration (mg/dL) and hematocrit fraction (%) are used to normalize analytically measured concentrations of any chemical substance measured in the hemolyzates to actual concentrations in the red blod cells.

For the hemocytometric characterization of each fresh blood sample, use the 150-m µL whole blood sample that was prepared as step (c) of the whole blood fractionation procedure. For field studies, this measurement is performed on as fresh as possible whole blood, if similar equipment is available at the site of sample collection.

That shown is a typical report with indicative values of standard deviation and relative standard deviation for a quintuplicate measurement of the sample Results from the results collection sheet are input to the calculation spreadsheet

MW(hHB)=64.454	**Name**	**Subj_ID**	**SD**	**CV**
day	**Date**	25/05/2021		
10^6_n/uL	**WBC**	9,0	0,2	2,2
10^6_n/uL	**LYM**	2,2	0,1	3,8
10^6_n/uL	**GRAN**	5,7	0,1	2,3
g/dL_WB	**HGB**	15,2	0,8	5,2
g/dL_RBC	**MCH**	33,4	0,3	0,8
HbcorrHt_g/dL	**MCHC**	33,8	0,3	1,0
10^6_n/uL	**RBC**	4,5	0,3	5,6
fL=10^-12 L	**MCV**	98,9	0,4	0,4
% v/v	**HCT**	44,9	2,7	5,9
%	**RDW%**	14,9	0,1	0,9
10^6_n/uL	**PLT**	261,2	10,5	4,0
10^6_n/uL	**MPV**	8,4	0,1	1,0
HbcorrHt_g/dL	HGB/HCT	33,7	7,9	23%
HbcorrHt_mM		5,2	1,2	23%

Note.

This procedure was optimized in the frame of wide-range studies where multiple biomarkers are measured in the same blood sample. In our hands, recovery of red blood cells from blood withdrawn in tubes containing polymeric gel for serum separation yielded very poor MALDI spectra.

Operative checklist for the measurement of glutathionyl-hemoglobin by MALDI-ToF mass spectrometry.

Preparation of standards and reagents.

Preparation of standards.

Hemoglobin. Accurately weight an approx. 2.5 mg sample in a 2-mL Eppendorf tube and dilute with deionized water to a final concentration 40 microM. Dilute appropriate volumes to 20, 10, and 5 microM (e.g., 0.5 mL solution + 0.5 mL water) with deionized water for spectrophotometric measurement. The dilution table is as follows.

a) Weight of human Hemoglobin standard: 2.5 mg
b) Amount of human Hemoglobin (micromoles): 10^6 * Weight / 64.454 = 38.78
c) Volume of water (mL) to obtain a 40 microM Hb solution: 40 / (b) = 0.970

Take 0.5 mL of solution (c) and dilute with 0.5 mL of water (solution (d), 20 microM)
Take 0.5 mL of solution (d) and dilute with 0.5 mL of water (solution (e), 10 microM)
Take 0.5 mL of solution(e) and dilute with 0.5 mL of water (solution (f), 5 microM)

Use solutions (c) – (f) for the standard curve of Plate C (see below)

Preparation of reagents (ingredients for MALDI matrix).

Trifluoroacetic Acid (1% stock solution). In a 50-mL dark glass bottle with polyethylene stopper, dispense 30 mL of chromatography-grade water and 300 microL of Trifluoroacetic Acid, (Sigma-Aldrich, 80457, eluent additive for LC-MS, LiChropur). Mix the contents by gentle inversion of the tightly stoppered container and store at +4°C. The mixture is functionally stable over 6-12 month.

Trifluoroacetic Acid (0.1% working solution). For the batch of 96 samples, dispense in a 1-mL Eppendorf tube 0,9 mL of chromatography-grade water and 100 microL of Trifluoroacetic Acid (1% stock solution, see above). Mix the contents by gentle inversion of the tightly stoppered container and store at +4°C. The mixture is functionally stable over the working day.

Sinapinic Acid. Weight powder in 1-mL Eppendorff tubes with acute cone bottom and retention hook for the lid. For the batch of 24 samples, weight approx. 8-10 mg (MW 259.18) and annotate weight to first 0.1 mg with solid felt-tip pen. To save time, prepare several batches of dry powder, appropriate for 24, 48 and 96-sample batches (depending on expected working schedules) and store them at room temperature in a Tupperware container lined with aluminum sheet to keep in the dark.

Combined Acetonitrile-Trifluoroacetic Acid solvent. For the batch of 24 samples, mix 0.5 mL of Trifluoroacetic Acid (0.1% working solution) and 0.5 mL of Acetonitrile, chromatography grade. Mix the contents by gentle inversion of the tightly stoppered container and keep at room temperature. The mixture is functionally stable over the working day.

Operative checklist for the measurement of glutathionyl-hemoglobin in red blood cells.

The procedure entails four main steps:

a) **Preparation of primary hemolysate**
b) **Titration of the primary hemolysate**
c) **Preparation of secondary titrated hemolysate by dilution of the primary hemolisate**
d) **Preparation of sample for MALDI analysis**
e) **MALDI-ToF analysis**

Labelling of sample plasticware. For each batch of samples, for each sample, the following sample containers and plasticware should be previously prepared and labelled.

1 x 1-mL Eppendorf tube with 1 mL of chilled sterile water	Preparation of primary hemolysate
1 x 1-mL Eppendorf tube with 1 mL of chilled sterile water	Preparation of secondary titrated hemolysate
2 x 0.2-mL Eppendorf tubes (PCR-type, with attached lids)	Preparation of MALDI sample

a) ***Preparation of the primary hemolysate from stored red blood cells***. The employed RBCs derive from a 50-microL aliquot obtained at the time of whole blood fractionation (see above).

Thaw the appropriate number of sample tubes. From each tube, deliver approx. 10 µL of separated RBCs into the first 1-mL Eppendorf tube with 1 mL of chilled sterile water. Stopper the tube and mix by inversion to completely hemolyze and resuspend the contents. Keep in tube holder on ice until all preparation is ended.

b) ***Titration of the primary hemolysate.*** Prepare a 96-well reading plate according to the scheme below.
Use the template A of the spreadsheet to keep track of operations and perform calculations.

Plate A(1): measurement of primary hemolysate

	1	2	3	4	5	6	7	8	9	10	11	12
**A**	O5	Ov	O1	O2	O3	O4	O5	O6	O7	O8	O9	O10
**B**	O5	Ov	O11	O12	O13	O14	O15	O16	O17	O18	O19	O20
**C**	O10	Ov	O21	O22	O23	O24	O25	O26	O27	O28	O29	O30
**D**	O10	Ov	O31	O32	O33	O34	O35	O36	O37	O38	O39	O40
**E**	O20	Ov	O41	O42	O43	O44	O45	O46	O47	O48	O49	O50
**F**	O20	Ov	O51	O52	O53	O54	O55	O56	O57	O58	O59	O60
**G**	O40	Ov	O61	O62	O63	O64	O65	O66	O67	O68	O69	O70
**H**	O0	O0	O71	O72	O73	O74	O5	O5	O10	O10	O20	O20

Legend.

O: Hb calibrator (µM)	On: sample (1-24)	

In the 96-well plate, for each well, the following volumes of sample and reagents are dispensed.

a) 200 µL of water based hemoglobin calibration solution is pipetted (2 x 40-20-10-5 microM) in wells 1H – 12H.
b) 200 µL of sample in wells 1A-12B (24 samples)
c) The plate is then inserted into the reader for spectrophotometric measurement at 450 nm (filter bandpass 8.5-16 nm). Absorbance measurements are transferred to the results collection sheet and input to the calculation spreadsheet. Depending on the specific plate reader used, analogic or digital data transfer can be employed.

The calculation spreadsheet yields the dilution factor of the primary hemolysate for the preparation of the secondary hemolysates at a constant hemoglobin concentration of 10 microM. The results is, for each sample, the number of microliters of primary hemolysate that should be mixed in 1 mL of secondary solution to yield such concentration.

c) ***Preparation of secondary titrated hemolysates by dilution of the primary hemolisates.*** Use the second set of 1-mL Eppendorf tube with 1 mL of chilled sterile water (see above).

For each sample, withdraw the number of microliters of water specified in template A of the spreadsheet from the 1-mL Eppendorf tube and immediately add the same volume of the primary hemolisate. Stopper the tube and mix by inversion to completely mix the contents. Keep in tube holder on ice until all preparation is ended.

Plate A(2): concentration check of secondary hemolysate

	1	2	3	4	5	6	7	8	9	10	11	12
**A**	O5	Ov	O1	O2	O3	O4	O5	O6	O7	O8	O9	O10
**B**	O5	Ov	O11	O12	O13	O14	O15	O16	O17	O18	O19	O20
**C**	O10	Ov	O21	O22	O23	O24	O25	O26	O27	O28	O29	O30
**D**	O10	Ov	O31	O32	O33	O34	O35	O36	O37	O38	O39	O40
**E**	O20	Ov	O41	O42	O43	O44	O45	O46	O47	O48	O49	O50
**F**	O20	Ov	O51	O52	O53	O54	O55	O56	O57	O58	O59	O60
**G**	O40	Ov	O61	O62	O63	O64	O65	O66	O67	O68	O69	O70
**H**	O0	O0	O71	O72	O73	O74	O5	O5	O10	O10	O20	O20

Legend.

O: Hb calibrator (µM)	On: sample (1-24)	

In the 96-well plate, the following volumes of sample and reagents are dispensed in each well.

a) 200 µL of sample in wells 1D-12E (24 samples)
b) The plate is then inserted into the reader for spectrophotometric measurement at 450 nm (filter bandpass 8.5-16 nm). Absorbance measurements are transferred to the results collection sheet and input to the calculation spreadsheet. Depending on the specific plate reader used, analogic or digital data transfer can be employed.

The calculation spreadsheet yields the final hemoglobin concentration in the secondary hemolisate, that should be close to the target value of 10 microM. Although the accuracy of this concentration is not crucial to the results of the MALDI measurement, excessively lower or higher values may yield excessive loss of precision.

d) Preparation of samples for MALDI analysis. Use the two sets of 0.2-mL Eppendorf tubes (PCR-type, with attached lids) (see above).
For each sample, withdraw 10 microL of secondary titrated hemolysate and dispense on the bottom of the labelled 0.2-mL PCR tube. Stopper the tube and put in storage rack (kept in its transport Tupperware box) at -20°C . These samples are used for MALDI analysis.

Absorbance measurements are analogically transferred to the results collection sheet and input to the calculation spreadsheet.

e) MALDI-ToF analysis. The prepared samples are transferred to the mass spectrometry laboratory for MALDI-ToF analysis.
Instructions are given for the specific instrument and software used. The instrumenti is an Autoflex III (Bruker Daltonics, Bremen, Germany) Matrix-Assisted Laser-Desorption Time-of-Flight (MALDI-ToF) mass spectrometer, equipped with a 355 nm Smart Beam solid state Nd:YAG UV laser and a 396-well plate sample holder. Software operation refers to the xxx version (Microsoft Windows XP operating system). Operators of other instruments may adapt each working phase to the requirements of their own devices.

Preparation of equipment and consumables. The following equipment is needed at the mass spectrometry facility.

10-microL single-channel pipetting device and matching tips.
2-microL single-channel pipetting device and matching tips.
Vortex mixer
Mini centrifuge.
Instrument's own MALDI sample plate and holder (Bruker MTP384 S/N 03229).

Prepare the following solutions (see above).

Trifluoroacetic Acid (0.1% working solution). For the batch of 24 samples, dispense in a 1-mL Eppendorf tube 0,9 mL of chromatography-grade water and 100 microL of Trifluoroacetic Acid (1% stock solution, see above). Mix the contents by gentle inversion of the tightly stoppered container and store at +4°C. The mixture is functionally stable over the working day.
Combined Acetonitrile-Trifluoroacetic Acid solvent. For the batch of 24 samples, mix 0.5 mL of Trifluoroacetic Acid (0.1% working solution) and 0.5 mL of Acetonitrile, chromatography grade. Mix the contents by gentle inversion of the tightly stoppered container and keep at room temperature. The mixture is functionally stable over the working day.
Sinapinic Acid. For the batch of 24 samples, take one 8-10 mg (MW 259.18) pre-weighted sample. From the weight annotated on the tube, calculate the volume of Combined Acetonitrile-Trifluoroacetic Acid solvent necessary to yield a 100 mM SA solution. A tested calculation module is available in worksheet A of the available spreadsheet suite. Dispense

the appropriate volume of solvent in the tube, stopper tightly and dissolve the solids by repeated vortexing (30-s cycle with 1-min rest: in all operations that involve the vortex mixer, the operator should check hands for signs of excessive exposure to vibration, such as itching and discoloring of fingertips).
Keep all equipment at hand. Print and have at hand the sample identification list.

Dispense in each of sample-containing 0.2-mL Eppendorf tubes (PCR-type, with attached lids) 10 microL of the SA solution (place the droplet on the side of the tube and let it drop to the bottom). Stopper the tube and keep the tube in holder at room temperature until all preparation is ended.
Vortex-mix for 30s a pair of sample tubes, then centrifuge one tube lot corresponding to the mini-centrifuge holding capacity to settle the liquid on the bottom. Never open the tube after dispensing the matrix solution.
When all the batch tubes are mixed and centrifuged, start the critical phase of sample deposition.

MALDI plate: sample deposition

The sketch below shows four of the 16 rows of the stainless steel sample plate of the employed instrument. It is held on the matching sample plate holder.

	1	2	3	4	5	6	7	8	9	10	11	12	13	14	15	16	17	18	19	20	21	22	23	24
**A**	○	○	○	○	○	○	○	○	○	○	○	○	○	○	○	○	○	○	○	○	○	○	○	○
**B**	○	○	○	○	○	○	○	○	○	○	○	○	○	○	○	○	○	○	○	○	○	○	○	○
**C**	○	○	○	○	○	○	○	○	○	○	○	○	●	○	○	○	○	○	○	○	○	○	○	○
**D**	○	○	○	○	○	○	○	○	○	○	○	○	○	○	○	○	○	○	○	○	○	○	○	○

Legend.

○: four replicate depositions of an individual sample (1 microL)

●: sample spot used for pre-run instrument checks (example)

The surface of the sample plate should be cleaned after each use to remove the preceding samples. The operator should wear nitrile gloves and touch the plate only through the sides of the holder. Hot tap water is sufficient to remove most of the material without the need of organic solvents. A gentle rub with a clean paper towel (white, non-dyed toilet paper fits the need and can be discarded as sanitary waste). Next, small volumes of distilled water are poured and gently frictioned with a paper towel, until evaporation leaves a shining surface. Last, a small volume of matrix solvent is poured and gently frictioned with a paper towel, until evaporation leaves a shining surface, ready for sample deposition.

Each sample is deposited from its prepared sample-matrix tube in four 1-microL aliquots, each placed in one separate spot of the plate, in vertical columns. Use the sample identification list to check and keep track of the correspondence of samples and loading positions. Loading of each sample in four spots takes approx. 90-120s (this Operator's pace). A technique tip is to dispense first a very small fraction of the 1-microL solution, gently rubbing around the spot surface, then dispense all the rest and gently mix on the surface. A favorable sign is some feedback sensation of rubbing ("scratching") the steel with the plastic tip (over 10 years of using the same plate did not cause any scratching sign on the surface, visible under a magnifying glass). It is (very!) detrimental to the final measurement that dried spots are touched any more. Use the plastic lid and store the plate in the transport box if it is left unattended.

MALDI analysis: sample description in the automated acquisition software

The prepared, naturally air dried sample plate is inserted in the mass spectrometer through its vacuum lock, which operates in an automated pressure-feedback cycle. It is detrimental to long-term instrument performance that final drying is performed in the vacuum lock.

The sample working list is generated in the appropriate section of the instrument's software to allow automated analysis.

MALDI analysis: sample plate loading and preliminary instrument regulations

Once the sample plate is loaded and automated instrument routine checks are complete, some preliminary instrument regulations can be checked. One or two sample spots (●) can be used if the instrument is routinely maintained, since the "true" sample analyses on the same spot are usually not influenced, if only minor adjustment is necessary.

Observation of sample deposition. A few spots can be observed through the instrument micro-camera to ensure that there are no gross irregularities in surface texture. At this time, no remedy is possible, but chance for sample failure can be taken into account to explain final results.

Instrument operation. The instrument is operated according to the manufacturer's directions in the linear positive ion mode at a calculated resolution of approximately 1000.

Laser shot fluency. Laser power is set at such value (as percent fraction of its full power) that peak intensity of the alpha-Hb chain (the most intense one) would be between 500-1000 cps in successful individual shots, with a minimum final height of approx. 2500-5000 cps.

Calibration of mass axis. At the beginning of each session, internal calibration of the m/z scale is accomplished in the random sample spot used for the preliminary instrument regulations, by using the signals of doubly- and singly-protonated homozygous alpha- and beta-hemoglobin (m/z 7564.2; 7934.2; 15128.4 and 15867.5 respectively). This calibration is stored in the instrument digital logbook.

MALDI analysis: automated sample analysis

A final check is performed by running an automated analysis of a single spot after all preliminary instrument regulations are performed. After each laser shot, detection of a signal of a-Hb higher than 500 cps prompts the FlexControl™ software to add the raw spectrum to the integration register, until a number of 50 valid spectra are obtained on the sample spot and analysis is considered complete. The obtained spectrum is used to check or update mass calibration for the corresponding analysis batch

Once the preliminary instrument regulations are successfully performed, the automated analysis routine is started and proceeds unattended. Measurement on an individual spot takes approximately 45-60 sec. The measurement of a 24-samples batch (96 individual measurements) is complete within approx. 2 hours.

It is detrimental to long-term instrument performance that the sample plate remains in the instrument after analysis is complete, because sublimation of the MALDI matrix in the source high vacuum contaminates the inner walls without reason. Therefore, after the analysis is complete, the instrument control software displays a sample plate map where green dots correspond to positively analyzed samples, orange ones to those that need manual check and white to spot positions where the automated acquisition routine could not detect valid signal and laser shooting was thus aborted. The Operator can now repeat the measurement of aborted samples manually, until a satisfactory result or complete spot failure is achieved.

MALDI analysis: post-acquisition sample processing

The batch analysis file is downloaded from the instrument for off-site data analysis. This procedure entails four phases, as follows.

a) Calibration and annotation of individual spectra with the FlexAnalysis™ software

b) Export of sample name-coded binary mass-intensity .txt files and of calibrated peak lists for each individual spectrum as an Excel spreadsheet. The analysis of each sample entails four separate sets of data files.

c) Import of each quartet of name-coded binary mass-intensity .txt files in the spreadsheet-based data analysis tool and operation to obtain the percent glutathionyl-hemoglobin fraction, as percent, with associated standard uncertainty.

d) Preparation of final analytical report of sample batch with associated sample identity information.

Individual post-acquisition phases are performed by the Operator as follows.

a) ***Calibration and annotation of individual spectra with the FlexAnalysis™ software.*** The four analysis files that are obtained for each blood sample (four deposited spots, see above) are opened in the FlexAnalysis™ software and each is individually calibrated through the four main signals of doubly- and singly- protonated homozygous alpha- and beta-hemoglobin (m/z 7564.2; 7934.2; 15128.4 and 15867.5 respectively) that are contained in the specific calibration list. No preliminary smoothing or baseline subtraction function is applied to the spectra prior to the calibration command.

b) ***Export of sample name-coded binary mass-intensity .txt files and of calibrated peak lists.*** A name-coded directory in the data transfer device is prepared to host the data files for further elaboration. Once the post-acquisition calibration of each of the four spectra of each blood sample are performed, the corresponding .txt files are exported and saved into the name-coded directory. The generated mass-peak list is exported as the matching sample name-coded Excel spreadsheet file, with each of the four analysis spots reported in one spreadsheet page.

c) ***Import of name-coded binary mass-intensity .txt files in the spreadsheet-based data analysis tool.*** By using the import function of the Excel software, each .txt mass-intensity file is imported into the spreadsheet template, which is saved in the sample name-matching directory. Filenames are copied-and-pasted in the appropriate results table spreadsheet page.

   Simultaneous elaboration of the four spectra is performed automatically as follows.

   1) All four mass-intensity profiles are plotted together (four different colors) in three consecutive scatter graphs of the Excel spreadsheet, one as “raw-data (**a**)”, one as “raw-data baseline subtracted (**b**)”, one as “normalized intensity baseline subtracted (**c**)” (see Figure). The main molecular species of human hemoglobin in the MALDI-ToF spectrum are identified in the m/z axis by comparison with the calculated values of the corresponding profiles, which are reported in an auxiliary spreadsheet page. The profiles yield the integration boundaries of the corresponding species on the x-axis (m/z) to calculate peak area and signal intensity.

The three consecutive scatter graphs of the Excel spreadsheet, one as "raw-data (**a**)", one as "raw-data baseline subtracted (**b**)", one as "normalized intensity baseline subtracted (**c**)" are reported below for the example of a measurement that yields the following results.

		rep	HbSSG%	ES	HbGlc%	ES
	Sample_name	4	int_BL		int_BL	
**22Sep_co130**	**22Sep_co130**	**media**	**4,9%**	**0,1%**	**10,1%**	**0,3%**
22Sep_co130	**22Sep_co130_0_M14_1**	1	***5,0%***		***9,7%***	
22Sep_co130	**22Sep_co130_0_N14_1**	2	***4,8%***		***10,1%***	
22Sep_co130	**22Sep_co130_0_O14_1**	3	***4,9%***		***10,3%***	
22Sep_co130	**22Sep_co130_0_P14_1**	4	***4,9%***		***10,4%***	

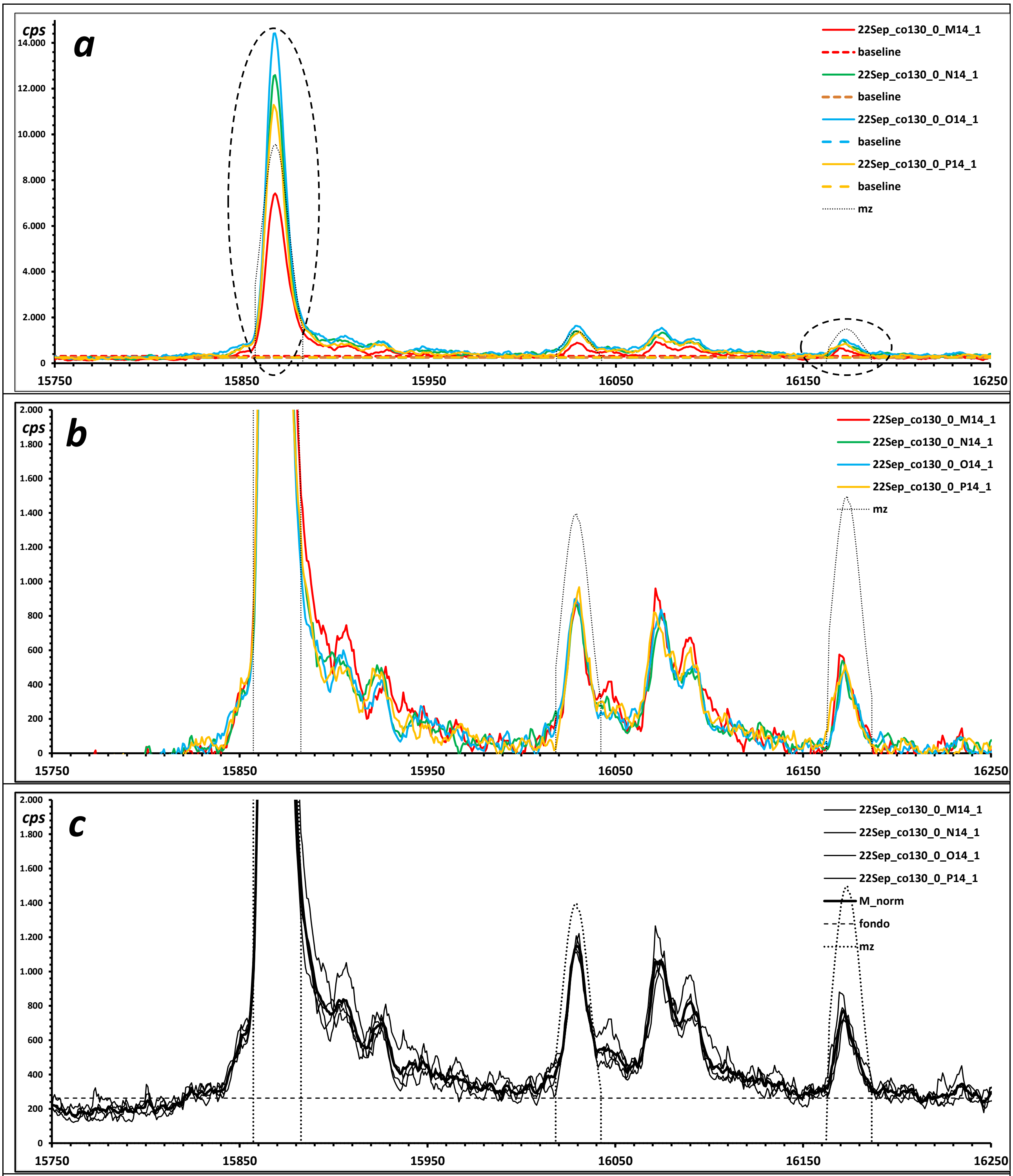

The three consecutive scatter graphs of the Excel spreadsheet, one as "raw-data (**a**)", one as "raw-data baseline subtracted (**b**)", one as "normalized intensity baseline subtracted (**c**)".

2) A provisional baseline is automatically calculated as the median intensity value throughout the spectrum, and plotted in the scatter graphs. The reported baseline color matches the corresponding spectrum (scatterplot ***a***).

3) The spreadsheet automatically normalizes the intensity of each intensity-vs-m/z profile to the intensity of the most intense signal (usually that of the alpha-hemoglobin MH^+ species) and calculates the corresponding baseline for subtraction (scatterplot ***c***).

4) The Operator modulates the baseline multiplication factor of each spectrum, in order that the actual displayed baseline adequately crosses the signal variability close to the profiles to be integrated (scatterplot ***c***). the Operator checks the effect of baseline modulation on the raw spectrum (scatterplot ***b***). Simultaneously, the spreadsheet integrates the underlying peak area to yield the corresponding signal intensity.

5) The spreadsheet automatically calculates, for each of the four spectra, the peak area ratio of glutathionyl-hemoglobin (centered around m/z 16,174) to beta-hemoglobin (centered around m/z 15,868) as percent value and reports the individual results in the sample report page. The spreadsheet calculates a mean percent value and the associated standard error. The same procedure is applied to the measurement of glycated beta-hemoglobin (centered around m/z 16,030) in the same sample, the relative level of which is employed to calculate the "glycated-corrected" fraction of HbSSG in the final report.

6) The Operator separately modulates the baseline multiplication factor of each spectrum in order to minimize the standard error of the quadruplicate determination, by visually aligning the four profiles in the "normalized intensity baseline subtracted" scatterplot graph.

d) ***Preparation of the final analytical report of sample batch.*** Results from the sample report page of each determination are sequentially copied into the analytical report page to prepare the final report. The level of HbSSG is reported as the "raw" ratio of HbSSG/b-Hb and as the "glycated corrected" ratio, as HbSSG/(b-Hb + Glc-b-Hb).

The attached suite of template spreadsheets contains the employed spreadsheets without any data and a separate example file with a quartet of mass-intensity profiles that can be used as exercise to gain familiarity with the tool.
